# Social Integration as Mediator and Age as Moderator in Social Capital Affecting Mental Health of Internal Migrant Workers: A Multi-Group Structural Equation Modeling Approach

**DOI:** 10.3389/fpubh.2022.865061

**Published:** 2022-05-12

**Authors:** Jingjing Zhou, Jianfang Zhou, Hongyang Zhang, Junwei Zhang

**Affiliations:** ^1^School of Sociology and Population Studies, Nanjing University of Posts and Telecommunications, Nanjing, China; ^2^School of Population Studies, Nanjing University of Posts and Telecommunications, Nanjing, China; ^3^Department of Sociology, School of Social Sciences, Tsinghua University, Beijing, China; ^4^College for Philosophy and Political Sciences, Shanghai Normal University, Shanghai, China

**Keywords:** social capital, social integration, depression, age, migrant workers, China

## Abstract

The rise of migrant workers has been a unique social phenomenon as China goes through industrialization, urbanization, and modernization. They are a special social group formed during the economic and social transition of the country. Migration of rural labor has pushed China on its new path toward industrialization and urbanization. Because of the urban-rural dual system of the country, however, it is difficult for migrant workers to be fully integrated into host cities, making them susceptible to negative emotions and mental health issues. Therefore, their mental health is an issue of great volume in the domains of social undertakings, people's livelihood, and public health. However, existing studies have paid limited attention to the psychological profile of migrant workers and even less to the interplays among their social capital, social integration, and mental health. Targeting China's internal migrant workers, this article tapped the interactions among their social integration, social capital, and mental health with a sample of the cross-sectional data from the China Labor Dynamics Survey (CLDS) in 2018. Multi-group structural equation modeling (SEM) was employed to test the moderating action of age by analyzing whether the mediation model differed significantly in the paths among young, middle-aged, and older migrant workers. The SEM based on bootstrapping suggested that, after controlling for the influence of gender, education, marital status, personal annual income, employer type, and self-rated health, migrant workers' social capital positively affect their mental health in a significant way, with social integration playing a mediating role. In terms of age difference, middle-aged migrant workers were more subject to the aforementioned mechanism than young ones, and young migrant workers were more affected by the mechanism than older ones. This study revealed different psycho-social interplays among social capital, social integration, and mental health across young, middle-aged, and elderly migrant workers. The findings could serve as an important theoretical reference and as practical guidance for improving policies concerning migrant workers' mental health and social benefits in the context of economic transition.

## Introduction

Thanks to economic globalization, international migrant workers worldwide have counted 189 million, representing the vast majority of international migrants (1). In China, there are as many as 285 million migrant workers, also known as internal migrants for work (2). They are a special social group formed in, and main contributor to, China's industrialization, urbanization, and modernization, and the rise of this class of workers is a unique social scene over the course of China's economic and social transformation (3). Generally speaking, migrant workers are workers born in rural areas, migrated to towns or cities to engage in non-agricultural work, but still have rural *hukou* or registered permanent residence (4). However, due to the segregation of cities and the countryside in China and the restrictions established by the household registration system, migrant workers can hardly obtain citizenship and the same social benefits as the locals (5). In addition, because of the large gaps in human capital and economic capital from the locals, most migrant workers are employed in the secondary labor market and live in urban fringe areas, making them susceptible to negative emotions and mental health issues (6).

Generally, personal endowment, migration distance, migration pattern, and social environment are regarded as the main factors affecting migrants' mental health (7). However, in the context of social transformation in China, frequent migration and mobility have brought about the reconstruction and transformation of such social capital as social networks and social trust of migrant workers, which directly affects their perception and digestion of negative emotions (8). In recent years, how social capital acts on mental health has been subject to extensive discussion in academic circles (9). Besides, in the absence of institutional support for citizenization and the deficiency in personal endowment of migrant workers, social capital embedded in social networks can effectively reduce the economic cost, psychological cost, and various risks in the process of migration; this helps their fusion with the local environment in economic, cultural, psychological, and identity terms (10). In the light of social capital promoting social integration, the social inclusion of migrant workers further improves their mental health by improving the sense of belonging and subjective wellbeing (11). This provides an empirical indication that social integration serves as a mediator in social capital acting on the mental health among internal migrant workers in the Chinese context (12).

In addition, large-scale migration of people from rural areas to cities for work has continued for more than 30 years in China (13). Along with social transformation and changes, the social differentiation within the migrant worker group has intensified, which is prominently manifested in the intergenerational differentiation between the elderly migrant workers (i.e., the first-generation migrant workers) and the middle-aged and young migrant workers (i.e., the new-generation migrant workers) (14). Significant differences exist among the three migrant worker subgroups when it comes to social capital, social integration, and mental health (15); however, research on the mechanism of action among migrant workers' social capital, social integration, and mental health has rarely combined with the age difference of migrant workers (16). Therefore, targeting migrant workers in China, this article gleaned the cross-sectional data from the 2018 China Labor Dynamics Survey (CLDS) to probe the links among social capital, social integration, and mental health, to examine how social integration acts as a mediator in social capital acting on mental health, and to conduct multi-group testing of age differences in the above mechanism with view to providing theoretical insight and practical guidance for improving policies concerning migrant workers' mental health and social benefits in the context of economic transformation.

## Literature Review

### Social Capital and Mental Health

Divided into individual and collective levels, social capital from an individual's perspective refers to the resources embedded in the individual's social relationship network, while collectively, it entails the trust, reciprocity, and norms within a group (17). Numerous empirical studies have proposed that for migrant workers social capital can significantly improve their mental health (18), which has been applied to mental health promotion programs (19). Using the stress process model, some scholars pointed out that social capital can alleviate perceived stress and buffer the negative impact of life events, and thereby promote mental health (20). Some scholars have noted how community-based social capital acts on the mental health, arguing that community-based cognitive social capital can promote mutual trust between migrants and local residents, eliminate the state of exclusion among floating populations, and create a good atmosphere for the community (21). However, divergence persists in academic circles about how social capital affects mental health (22). Some scholars opined that cognitive social capital (social trust, for instance) and mental health are positively correlated, but that structural social capital (social network, for instance) is not significantly correlated with mental health (23).

### Social Capital and Social Integration

Social integration or inclusion is an interactive and multi-dimensional dynamic process. Social capital can help migrant workers obtain resources from social networks that deliver instrumental or emotional support, while social trust helps them to achieve physical goals (24). At present, that the social capital positively acts on the social inclusion of migrant workers has been widely discussed in academic circles (25). The positive influence of migrant workers' social capital on their social integration has been demonstrated from the perspective of resource acquisition—i.e., migrant workers obtain valuable information, social support and material assistance through social capital to promote their social integration (26). For example, Lancee (27) found that migrant workers glean recruitment information (and, thus, job opportunities) through social networks to promote their economic integration. Other scholars discussed how social capital positively acted on the inclusion of migrant workers from the aspects of culture and identity—i.e., that social networking and social participation can help migrant workers form positive social connections with local residents, promote mutual understanding, and gain a sense of belonging and identity in host cities (28). However, some scholars have pointed out that over-reliance on ascribed social relationships in social networks may result in difficulties in social inclusion (29).

### Mental Health and Social Integration

In Chinese context, migrant workers' social integration or inclusion is defined as the reduction of their objective and subjective differences from urban citizens, so as to reduce the negative influence of social class inequality on mental health (30). A host of empirical studies have shown that a higher degree of social inclusion corresponds to better mental health (31, 32). Research in light of attachment theory has examined how social integration promoted mental health, finding that migrant workers with a higher degree of social inclusion enjoy a greater sense of group belonging and meaning in life, and hence lower possibility of depression (33). For example, Chen et al. (34) pointed out that migrant workers' strong attachment to the countryside may lead to lower satisfaction with their lives in the cities, but that with a higher degree of social integration, migrant workers will see their urban identity and sense of belonging significantly increase, hence better mental health. Other scholars have discussed how social integration positively acted on mental health from the viewpoint of social equality, arguing that migrant workers with a higher degree of social inclusion have a lower sense of relative deprivation, stronger sense of social equality, and better mental health (35).

### Social Capital, Social Integration and Mental Health

Plenty of studies have scrutinized how social capital and social integration each acts on mental health (36, 37). For example, Steel (38) pointed out that the more trusted friends migrants have, the higher their social trust, and the better their social integration is promoted, and that this improves self-rated health. However, there is a lack of well rounded and sharply defined research and empirical explanations on the mechanism of interplay among migrant workers' social capital, social integration and mental health. Furthermore, some scholars have posited that social capital does not necessarily positively affect mental health and its positive effect on mental health is more significant in a more harmonious social environment; this offers theoretical possibilities for discussing social inclusion to mediate the action of social capital on mental health (39). Other scholars have pointed out that to investigate how social capital affects mental health one needs to consider its applicable objects and that the perspectives of intergroup interaction and life course can help clarify the link between mental health and social capital (40).

### Age Difference

At present, Western scholars pay less attention to age difference in the associations among social capital, social integration, and mental health (41, 42). Some scholars have observed based on critical reviews on migrant health research that the introduction of a life course perspective helps clarify the distribution of the effect of social determinants on health among migrant populations (43, 44). With regards to the Chinese context, some scholars have noted intergenerational differences concerning social capital, social inclusion, and mental health of migrant workers, but most of them discuss the intergenerational differences from one single dimension and from the perspective of characteristics comparison (45). In China, elderly migrant workers (that is, the first-generation migrant workers) have relatively closed and homogenous social networks, mainly generated from blood, kinship, and geographical ties in rural areas, which thus implies a stronger sense of attachment to the countryside and a lower desire to integrate into the city than that of young and middle-aged ones (46). In the meantime, compared with middle-aged and elderly migrant workers, young migrant workers have a higher perception of social integration, and thus poor social integration is more likely to lead to a decline in their mental health (47). In addition, a small number of scholars have verified the intergenerational differences in the mechanism of action on migrant workers' mental health in combination with life course theory (48, 49). These studies provide empirical support to discuss intergenerational differences in the mechanism of action among migrant workers' social capital, social integration and mental health.

### Gaps in Existing Literature and Establishment of Current Hypotheses

**Figure 1:**
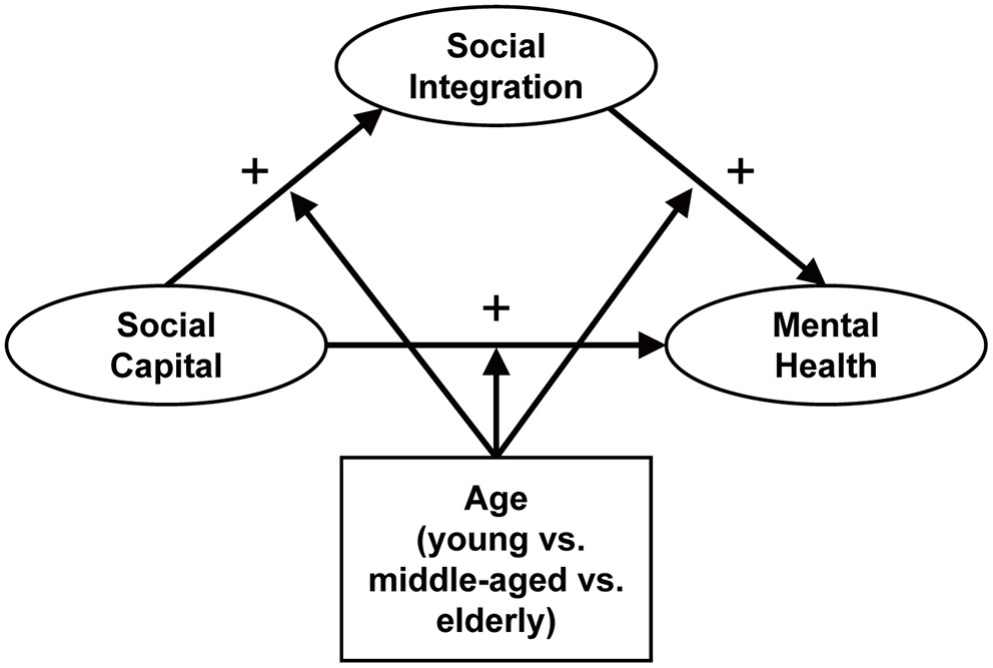
Conceptual framework.

Most of the existing research has examined how social capital and social inclusion each acts directly on the mental health for migrant workers (50, 51), or explains how social capital affects social integration among migrant workers (4). However, there has been no research offering a comprehensive exposition of the mechanism of interplay among migrant workers' social capital, social integration, and mental health. In addition, most of the existing research has discussed the intergenerational differences among migrant workers' social capital, social integration, and mental health from a single dimension (52). Whether the intensity of social capital and social integration acting on mental health differs among young, middle-aged, and elderly migrant workers has not been fully verified. Therefore, this study established a comprehensive conceptual framework to tap the interplays among social capital, social integration, and mental health status among internal migrant workers in China, as well as age differences therein (see Figure 1). Three core hypotheses were examined. H1: The higher the level of social capital, the better the mental health. H2: An increase in the level of social capital will improve social integration, thereby enhancing mental health. H3: How social capital acts on mental health through social integration may vary by age.

## Methods

### Data

This study sourced the data from the 2018 China Labor Dynamics Survey (CLDS). A comprehensive social survey in large scale, the CLDS is designed and implemented by the Center for Social Science Survey at Sun Yat-sen University in China. It adopts a multi-stage, multi-level, and probability proportional to size of labor force sampling method, covering the labor population aged 15–64 in 29 provinces, cities, and autonomous regions across the country. The survey was officially launched in 2012 and is carried out every 2 years. The latest survey was in 2018. This study defined migrant workers as 15–64-year-old non-agricultural laborers in urban areas with an agricultural household registration (53). After removing those with missing values for key variables—accounting for 1.8% of the population- the effective sample finally consisted of 8,346 individuals.

### Measures

#### Dependent Variable

The degree of depression was used herein to measure the mental health (54). Cai and Xu (55) questionnaire asked about the occurrence of 20 conditions in the past week: “I am troubled by some trivial stuff,” “I have not appetite to eat,” “Even if my family and friends help me, I still can't get rid of my depression,” “I feel I'm worse than most people,” “I'm depressed,” “I can't concentrate when I do things,” “I feel exhausted with everything,” “I feel hopeless,” “I think my life is a failure,” “My sleep quality is bad,” “I'm scared,” “I feel unhappy,” “I don't talk as much as usual,” “I feel lonely,” “I feel my life is boring,” “I feel people are not very kind to me,” “I used to cry,” “I feel apprehensive,” “I don't feel liked by people,” and “I don't think I can go about my daily work.” The frequency options were “1 = 5–7 days, 2 = 3–4 days, 3 = 1–2 days, and 4 = less than 1 day,” in which higher values indicated lower degrees of depression and hence better mental health.

#### Independent Variable

In the research on migrant workers, scholars often use Putnam's definition of social capital (56), arguing that social capital mainly consisted three parts (network, trust, and reciprocity) and divided into two types: cognitive social capital, including trust, reciprocity, etc., which is marked by pronounced individual subjective wishes, and structural social capital, including social participation, socializing frequency and pattern, etc., which is susceptible to objective conditions (57). Therefore, social capital was measured in this study from two aspects: social trust and social network. CLDS (2018) questionnaire divided social trust from the perspective of specific objects into nine categories. They were trust in, respectively, family members, neighbors, schoolmates, fellow villagers, strangers, colleagues, vendors, and religious believers, rated by level of trust from low to high (1 = completely untrustworthy, 5 = completely trustworthy). Regarding the social network, the questionnaire includes “How often does mutual help occur between you and your neighbors and other residents of this community?” with options on an ascending scale from 1 = never to 5 = always.

#### Mediating Variable

To measure social integration, most studies include such dimensions as economic inclusion, acculturation, identity fusion, and psychological inclusion (58), which may vary in denotation but are similar in connotation and measures (59). The present study applied the same division of social integration for migrant workers. Among them, economic integration refers to the integration in terms of employment, income and social security (60). It was measured by self-assessment of family economic status. The questionnaire asked the following question: “Generally speaking, how do you feel about the economic status of your family?” with options in such order from low to high as 1 = very dissatisfied to 5 = very satisfied. Cultural integration refers to familiarity with the language, lifestyle, and social customs of the inflow area and the degree of participation in cultural activities (61). It was measured by dialect proficiency. The following question was asked in the questionnaire: “How well have you mastered the local dialect?” (1 = none, 2 = little, 3 = partially, 4 = mostly, 5 = totally). Psychological integration refers to the psychological judgment of the degree of satisfaction and happiness in life in a city or town (62). It was measured by subjective wellbeing, and the following question was inquired: “Overall, how happy do you think your life is?” with the level of happiness assigned with values ranging from 1 = very unhappy and 5 = very happy. Identity integration refers to one's psychological distance from locals and fellow villagers, sense of belonging, and perception of where one is going in future. It was measured by whether to settle locally. The questionnaire asked the following question: “Are you likely to settle locally in the future?” with options in an ascending order from 1 = very unlikely to 5 = very likely).

#### Covariates

Seven demographic characteristics and two family elements constituted the covariates. The seven demographic variables were gender (0 = male, 1 = female); education (0 = none, 1 = primary school, 2 = junior high school, 3 = high school, 4 = bachelor's, 5 = master's); employer type: administration (0 = no, 1 = yes), enterprise or social organization (0 = no, 1 = yes), individual business (0 = no, 1 = yes), and freelance or non-regular job (0 = no, 1 = yes); and self-rated health (1 = very unhealthy, 5 = very healthy). The two family elements were marital status (0 = single, 1 = married) and personal annual income.

### Analytical Approach

SPSS 23.0 was used to organize data, sort out descriptive statistics before correlation analyses. According to the research purpose, Amos24.0 was used to establish and analyze the measurement model and the structural model among variables. Such indices as chi-square (χ^2^), comparative fit index (CFI), Tucker–Lewis index (TLI), root mean square error of approximation (RMSEA), and standardized residual mean square root (SRMR) were used to evaluate the goodness of the model-data fit. Specifically, non-significant chi-square values (*p* > 0.05), CFI and TLI above 0.90, and RMSEA and SRMR below 0.08 each denote a good fit (63). Bootstrapping was used to test the mediating effect (5,000 re-samples), in which the effect is deemed significant with the 95% confidence interval (CI) not including 0 (64). In addition, multi-group structural equation was used to evaluate the age difference in the overall model, in which the critical ratio of difference (CRD) was used to compare the structural path coefficients across groups. If the absolute value of the CRD is higher than 1.965, then there is an inter-group difference at the level of *p* < 0.05 (65).

## Results

### Descriptive Statistics and Correlation Analyses

The descriptive statistical results of the demographic variables herein are show in Table 1. Among the 8,346 migrant workers sampled, 39.5% were aged 31–50, which means that middle-aged migrant workers represented the largest proportion of the surveyed. Slightly more male migrant workers were surveyed than female ones (54.4%); most of the participants had finished middle school (38.9%); about four-fifths were married (81.4%); and they mostly worked for enterprises or social organizations (37.2%). In terms of self-rated health, most migrant workers had a good self-assessed health status (43.6%). As seen from Table 2, social capital, social inclusion, and mental health were significantly positively correlated, indicating a significant correlation among the key variables involved in this study, thereby satisfying the prerequisites of the mediation effect test (66).

**Table 1:** Descriptive statistics of variables (*N* = 8,346).

	**Frequency (n) or mean**	Percentage (%) or SD^*^
**Gender**		
Male	3,805	45.6%
Female	4,541	54.4%
**Age**		
15–30	2,042	24.5%
31–50	3,297	39.5%
51–64	3,007	36.0%
**Education**		
Illiterate	777	9.3%
Primary school	1,861	22.3%
Middle school	3,246	38.9%
High school	1,531	18.3%
Bachelor (junior college included)	905	10.8%
Master	26	0.3%
**Marital status**		
Single	1,550	18.6%
Married	6,796	81.4%
**Employer type**		
Administrative institutions	1,531	18.3%
Corporate or social organizations	3,104	37.2%
Individual business	1,705	20.4%
Freelance or no formal employment	2,006	24.0%
**Self-rated health**		
Very unhealthy	237	2.8%
Unhealthy	924	11.1%
Average	1,889	22.6%
Healthy	3,636	43.6%
Very healthy	1,660	19.9%
Personal annual income (logarithm)	10.2	0.9

* *SD, standard deviation*.

**Table 2:** Correlation analyses among key variables.

	**Mean**	**Standard deviation**	**1**	**2**	**3**
1. Social capital	3.341	0.581	1		
2. Social integration	3.428	0.842	0.155^***^	1	
3. Mental health	3.552	0.518	0.198^***^	0.257^***^	1

*** *p < 0.001*.

### Measurement Model

Results from testing the scales of social capital and social integration showed that the Cronbach's alpha coefficients of social capital and social integration were 0.706 and 0.820, respectively, both greater than 0.7, indicating a good reliability (67).

The adequacy of fit was evaluated with the confirmatory factor analysis (CFA). The model included two latent variables: social capital and social integration. Results showed a good model-data fit with chi-square = 22.749 (*p* < 0.01; *df* = 8), CFI = 0.999, and RMSEA = 0.015. All factor loadings of latent variables were significant at the *p* < 0.001 level, among which the factor loading ranges for social integration and social integration were 0.742–0.827 and 0.667–0.848, respectively. The CR values of the latent variables were 0.763 and 0.825, both >0.6, and the AVE values were 0.617 and 0.543, both >0.5, indicating acceptable convergent validity of the model. Furthermore, multi-group analyses showed that the model was invariant among migrant workers of different ages at the configural, metric, and scalar levels.

### Structural Model

**Figure 2:**
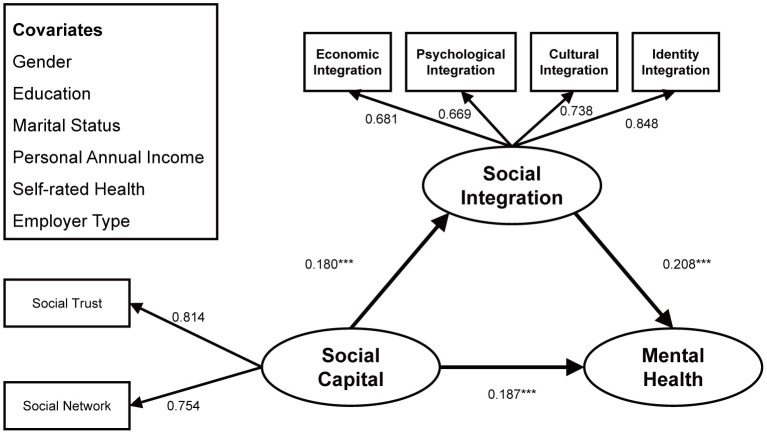
Standardized structural model (Full sample). ***p < 0.001.

The structural model had the fit indices: chi-square = 191.851, df = 44, *p* < 0.001, CFI = 0.994, TLI = 0.986, and RMSEA = 0.020, indicating a good model-data fit. The full-sample model as shown in Figure 2 and Table 3 suggested that social capital positively acted on the mental health in a significant way (β = 0.187, *p* < 0.001)—i.e., the higher the level of social capital, the better the mental health, confirming H1. Social integration mediated in an important way the link between social capital and mental health. Specifically, improved social capital contributed to higher degrees of social integration (β = 0.180, *p* < 0.001), thereby improving mental health (β = 0.208, *p* < 0.001) among migrant workers, confirming H2.

**Table 3:** Results of structural model for full sample and subsamples.

**Model paths**	**Full sample**			**15**–**30 years old**			**31**–**50 years old**			**51**–**64 years old**		
	**β**	**SE**	**CR**	**β**	**SE**	**CR**	**β**	**SE**	**CR**	**β**	**SE**	**CR**
Social integration ←-- Social capital	0.180^***^	0.016	12.909	0.145^***^	0.036	4.992	0.268^***^	0.027	12.754	0.085^***^	0.024	3.680
Mental health ←-- Social integration	0.208^***^	0.009	17.136	0.203^***^	0.017	8.161	0.265^***^	0.014	14.315	0.056^**^	0.016	2.796
Mental health ←-- Social capital	0.187^***^	0.011	14.828	0.14^***^	0.022	5.367	0.296^***^	0.018	15.317	0.064^**^	0.016	3.169
Mental health ←-- Gender	−0.032^**^	0.011	−3.029	−0.015	0.02	−0.675	0.006	0.019	0.34	−0.086^***^	0.018	−4.696
Mental health ←-- Education	0.036^**^	0.005	3.105	0.055^*^	0.011	2.436	0.032	0.009	1.867	0.018	0.009	0.950
Mental health ←-- Marital status	0.040^***^	0.015	3.578	0.008	0.022	0.33	0.074^***^	0.04	4.648	0.017	0.081	0.980
Mental health ←-- Personal annual income	0.044^***^	0.006	4.142	0.041	0.011	1.864	0.074^***^	0.01	4.445	0.035	0.009	1.946
Mental health ←-- Self-rated health	0.145^***^	0.006	13.234	0.101^***^	0.012	4.544	0.075^***^	0.01	4.653	0.268^***^	0.008	14.771
Mental health ←-- Individual business	−0.005	0.016	−0.372	0.043	0.032	1.463	−0.01	0.027	−0.544	−0.011	0.026	−0.571
Mental health ←-- Corporate or social organizations	0.021	0.014	1.562	0.042	0.029	1.352	0.035	0.024	1.784	0.002	0.021	0.090
Mental health ←-- Administrative institutions	0.026^*^	0.017	2.049	0.03	0.035	1.074	0.012	0.028	0.602	0.041^*^	0.025	2.060

*** *p < 0.001*,

** *p < 0.01*,

* *p < 0.05;*

*β, standardized coefficient; SE, standard error; CR, critical ratio*.

Among the covariates, mental health was better in male migrant workers than in female ones (β = −0.032, *p* < 0.01); those with higher education levels were more mentally healthy (β = 0.036, *p* < 0.01); compared with unmarried ones, married migrant workers had better mental health (β = 0.040, *p* < 0.001); a higher income corresponded to better mental health (β = 0.044, *p* < 0.001); better self-assessed health status indicated better mental health (β = 0.145, *p* < 0.001); migrant workers working in administrative or public institutions had better mental health than freelance or non-regularly employed ones (β = 0.025, *p* < 0.05), but other employer types had no significant correlation with the mental health. Overall, the full-sample model explained 9.2% of the variance in social integration and 14.3% of the variance in mental health status. The bootstrapping results are shown in Table 4. Social integration mediated the action of social capital on the mental health [β = 0.037, 95% bootstrap CI (0.03, 0.046)], accounting for 16.4% of the total effect [β = 0.225, 95%bootstrap CI (0.197, 0.252)], in which the indirect effect was indicated significant with the bootstrap 95% confidence interval not containing 0.

**Table 4:** Direct and indirect effects and 95% confidence intervals (CI).

**Model pathways**	**Full sample**			**15**–**30 years old**			**31**–**50 years old**			**51**–**64 years old**		
	**β**	**95% CI**		**β**	**95% CI**		**β**	**95% CI**		**β**	**95% CI**	
		**Lower**	**Upper**		**Lower**	**Upper**		**Lower**	**Upper**		**Lower**	**Upper**
**Total effect**												
Social capital → mental health	0.225	0.197	0.252	0.17	0.103	0.232	0.367	0.331	0.401	0.069	0.023	0.114
**Direct effect**												
Social capital → mental health	0.187	0.161	0.214	0.14	0.074	0.203	0.296	0.26	0.332	0.064	0.019	0.109
**Indirect effect**												
Social capital → social integration → mental health	0.037	0.03	0.046	0.029	0.016	0.047	0.071	0.055	0.088	0.005	0.001	0.011

*β, standardized coefficient*.

### Group Difference Test

Multi-group analyses in SEM were conducted to verify any significant difference in path coefficients due to age. First, the measurement model was found to be invariant (*p* > 0.05)—that is, the factor loadings were equal across age groups; secondly, we compared the unconstrained structural model where the structural path changed with age with the constrained one, in which the factor loadings, covariances, weights, residuals, etc. were set equal across young workers (15–30 years old), middle-aged migrant workers (31–50 years old) and elderly migrant workers (51–64 years old). The results showed a significant difference (*p* < 0.001) between the unconstrained model (χ^2^ = 324.563, df = 132) and the constrained one (χ^2^ = 682.186, df = 178).

The CRD tests showed significant divergences across young, middle-aged, and older migrant workers in the three pathways. First, significant divergences existed between young and middle-aged subgroups, between young and elderly subgroups, and between middle-aged and elderly subgroups in the path coefficient of social capital to mental health (CRD = −5.606, *p* < 0.05, CRD = 2.423, *p* < 0.001, and CRD = 9.265, *p* < 0.001, respectively). Secondly, significant divergences were seen in the path coefficient of social capital to social integration between young and middle-aged subgroups, between young and elderly subgroups, and between middle-aged and elderly subgroups (CRD = −3.761, *p* < 0.001, CRD = 2.136, *p* < 0.05, and CRD = 7.235, *p* < 0.001, respectively). Finally, significant divergences were seen in the path coefficient of social integration to mental health between young and middle-aged subgroups, between young and elderly subgroups, and between middle-aged and elderly subgroups (CRD = −2.481, *p* < 0.05, CRD = 4.096, *p* < 0.001, and CRD = 7.179, *p* < 0.001, respectively).

**Figure 3:**
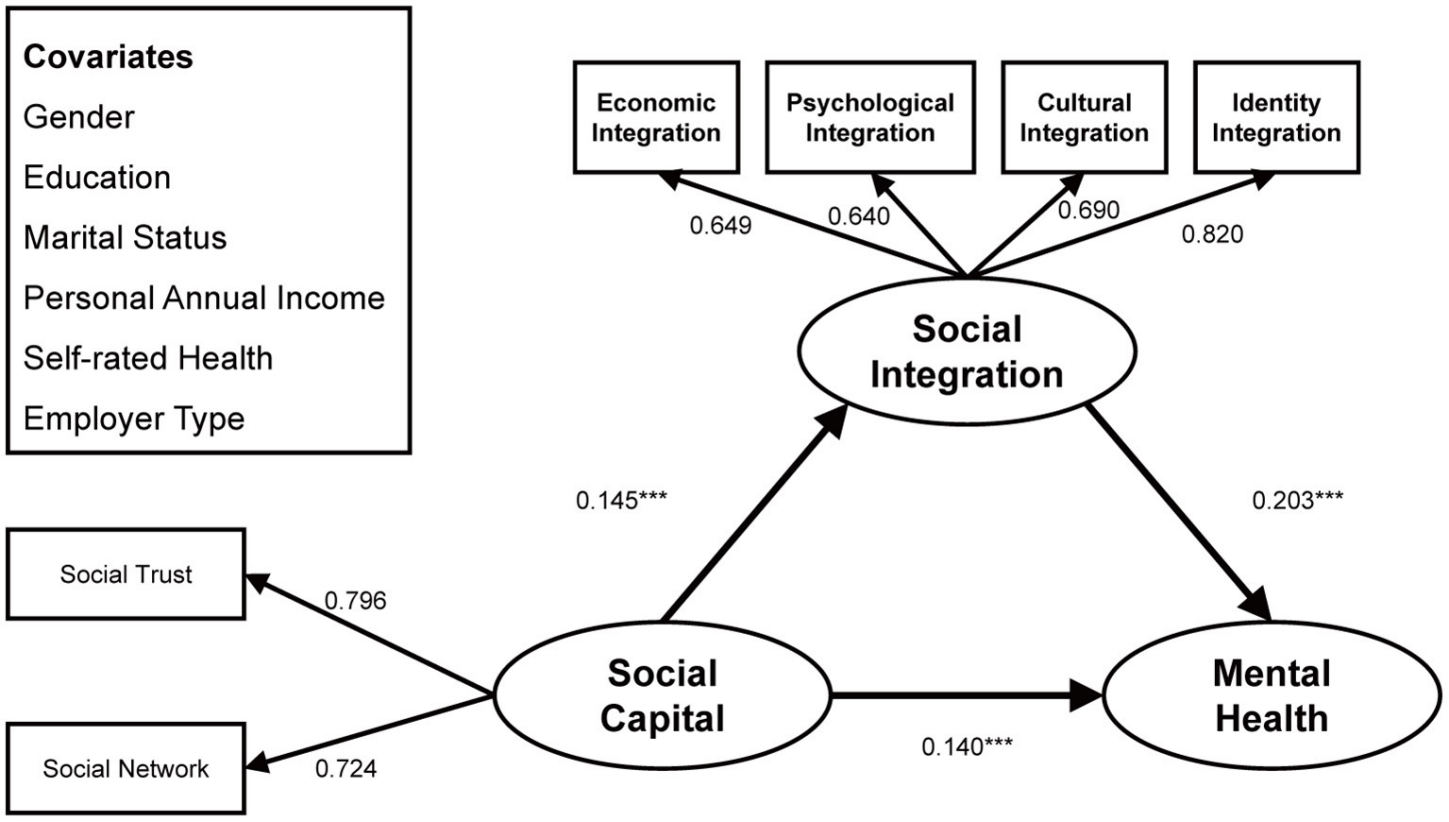
Standardized structural model (sub-sample: 15–30 years old). ***p < 0.001.

**Figure 4:**
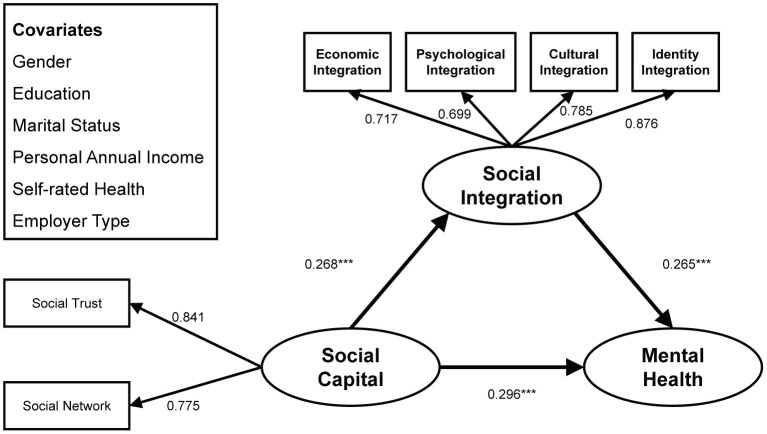
Standardized structural model (sub-sample: 31–50 years old). ***p < 0.001.

**Figure 5:**
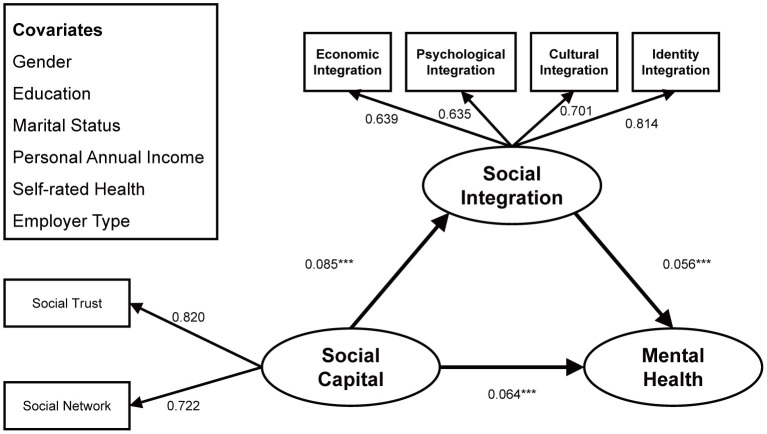
Standardized structural model (sub-sample: 51–64 years old). ***p < 0.001.

As shown in Figures 3–5 and Table 3, the above three structural paths had significant positive effects on young, middle-aged and elderly migrant workers, but the degree of effect varied. Specifically, on the path from social capital to mental health, the effect on the middle-aged subgroup (β = 0.296, *p* < 0.001) was more significant than that on the young subgroup (β = 0.140, *p* < 0.001), which in turn was greater than that on the elderly subgroup (β = 0.064, *p* < 0.001); on the path connecting social capital to social integration, the middle-aged subgroup (β = 0.268, *p* < 0.001) were more affected than the young subgroup (β = 0.145, *p* < 0.001), and the young subgroup was more affected than the elderly group (β = 0.085, *p* < 0.001); on the path connecting social capital to mental health, the middle-aged subgroup was more affected (β = 0.265, *p* < 0.001) than the young subgroup (β = 0.203, *p* < 0.001), who was more affected than the elderly subgroup (β = 0.056, *p* < 0.001).

## Discussion

Targeting migrant workers in the context of China's social transformation, this article investigated the interplays among their social capital, social inclusion/integration, and mental health, elucidated the mechanism of social integration mediating the way social capital acts on mental health, and provided theoretical support and coping strategies for bettering migrant workers' mental health.

The results show that enhanced social capital of migrant workers could effectively improve their mental health, a finding that supported H1 and kept with previous findings. Cognitive social capital (represented by trust) and structural social capital (represented by social network) are both significantly related to health status (68, 69). For migrant workers, improved social trust helps reduce the incidence of depression, and those with higher levels of social trust are better positioned to establish harmonious interpersonal relationships, which helps relieve tension and fear in urban life, and tend to have more positive psychological expectations when facing psychological difficulties, all of which significantly enhance their mental health (70). In terms of social network, on the one hand, migrant workers improve their economic capabilities by working, which can effectively resolve frictions and conflicts among family members, and can increase the sense of attachment between them, enhance the supportive role of the ascribed social network, and alleviate their mental pressure (71); on the other hand, the social network constructed and expanded by migrant workers in the city by working can effectively reduce the various pressures they face in the city, thereby curbing the psychological crisis (72).

Additionally, this study found that migrant workers with more social capital had a higher degree of social integration, thereby improving their mental health, which supported H2. This finding is considered an innovation in this study, which explains the interplays among social capital, social inclusion, and mental health of internal migrant workers by constructing a fresh framework. In China, where “social relations” are highly valued, social capital plays a more meaningful role than human capital (73). For migrant workers who lack both human capital and economic capital, their economic activities such as job hunting rely more on social network (74), so social capital promotes the economic inclusion of migrant workers. Besides, social capital provides an important way for migrant workers to participate in cultural activities, adapt to new urban lifestyles, and acquire a sense of identity, which supports achievement of expressive goals and enhances psychological integration and identity integration (75). On the basis that social capital promotes the improvement of social integration, social integration affects the mental health of migrant workers through health behavior influence paths, psychological influence paths, and physiological influence paths (76). For example, social integration can improve mental health by increasing individual control, sense of belonging, and generalized trust (77).

Multi-group analyses by age also provided valuable findings. The social integration-mediated action of social capital on mental health differed along age lines, a finding that supported H3. On the three paths by which social capital affected mental health, social capital affected social inclusion, and social inclusion affected mental health, middle-aged migrant workers (31–50 years old) were more affected than young migrant workers (15–30 years old), while the effect on young migrant workers exceeded that on older migrant workers (51–64 years old). Combined with the life course theory, when social changes have different effects on the cohorts of different generations, due to the obvious similarity in the experience of the same generation, the conditions of different generations tend to show significant differences, and therefore, the historical effect of life trajectories is manifested in the experiences of different generations (78). In China, the first generation of internal migrant workers (older migrant workers) emerged after the reform and opening up of the country, and the new generation (middle-aged and young migrant workers) emerged in the early 2000s. The new generation, compared with the first generation, has seen the economic and social situation undergo tremendous changes, leading to differences across the three subgroups in the mechanisms of interaction between social capital, social integration, and mental health (79). Compared with older migrant workers, middle-aged and young migrant workers generally have higher levels of education, higher occupational expectations, and more opportunities to move between urban and rural areas. With greatly improved social security and labor security systems, young and middle-aged migrant workers are in better positions than the elderly migrant workers with regards to both social network and social capital such as social trust, and enjoy a higher degree of multi-dimensional social integration in economy, psychology, identity, and culture (80). Compared with young ones, middle-aged migrant workers spend a longer time in the city and have richer work and life experience, which brings them more social capital, and thus a higher degree of social integration (42). In addition, due to aging and the decline of labor capacity among elderly migrant workers, except for a very small number of skilled and managerial talents who have become the backbone of an enterprise and thus stayed in the city, most of them have chosen to return to their hometowns to spend the rest of their lives. The lack of belonging to agricultural production and life makes young and middle-aged migrant workers eager to blend themselves with the cities and enjoy modern city life. Therefore, compared with the mental health of elderly ones, that of middle-aged and young migrant workers is more easily affected by the level of social capital and social integration (81).

This study has practical significance of value for bettering the mental health for migrant workers. The findings offer evidence supporting the government to improve the household registration system, build an integrated labor market, and establish supporting security systems such as healthcare, housing, children's education, and old-age care, providing institutional support for the social integration of young, middle-aged and elderly migrant workers and alleviate their feelings of being “marginalized” (82). On the other hand, companies where migrant workers are employed should establish mental health counseling and monitoring systems to give targeted psychological counseling and emotional regulation services for young, middle-aged, and elderly migrant workers. In pursuing work efficiency, the companies can organize employee assistance programs to elevate the human and social capital of migrant workers (83). In addition, each community should make full use of its resources, such as working with social organizations, increasing community activities, improving community conflict and dispute mediation mechanisms, etc. This would promote the acculturation and identity integration of migrant workers, help migrant workers and local residents form good social relations, and enhance the social trust, security, and happiness of migrant workers (84).

Objectively speaking, some gaps exist in the study that should be filled in follow-up research. First, although this study combined theory and SEM to tap the mechanisms of interaction between social capital, social integration, and mental health, because cross-sectional data was used, the establishment of causal relationships needs to be deepened with experimental and longitudinal design. Secondly, in addition to social integration as the mediating variable and demographical and family characteristics as the covariates selected herein, there may be other variables that play mediating roles in connecting social capital and mental health among internal migrant workers in combination with relevant literature, such as social status in subjective and objective terms, social support, etc. (85). Besides, the security status of migrant workers, such as the type of labor contract (permanent/casual), whether they have social insurance, employment security, etc., will also affect their health status (86). Therefore, future research can include more variables to conduct diversification analysis. Thirdly, combining the inter-group differentiation among internal migrants in China, i.e., age grouping, this study explored the inter-group differences in the mechanism of action among their social capital, social integration, and mental health. In addition to age, factors such as income and gender may also cause differences in the mechanism of association among related variables (87). The follow-up research may conduct multi-group difference analysis from other perspectives. Finally, due to the limitation of data, the measurement of the health status of the sample focused on mental health and self-assessed health with little attention to objective physical measures. Although self-rated health data effectively reflects the self-perception of individual health status, and has good reliability and validity in the Chinese social environment (88), it is still affected by cognitive biases of individuals, such as “feel too good about oneself,” answering biases, etc. Hence, in future research, both self-assessed and objective health indicators should be collected at the same time to ensure a holistic view of the research object and thereby arrive at more reliable conclusions.

## Conclusion

Targeting Chinese internal migrant workers with a sample from the CLDS database, this study revealed the mechanisms of interaction between their social inclusion, social capital and mental health and verified age differences therein. The research results showed that social capital positively affected mental health in a significant manner, with social integration playing a mediating role therein; middle-aged migrant workers were more affected by the above mechanism than young migrant workers, and the effect on young migrant workers exceeded that on the elderly migrant workers. Despite certain limitations, this study raises some intriguing questions about migrant workers in China, offering important theoretical reference and practical guidance for policies to better the mental health and social benefits for migrant workers with age considerations in the context of economic transition.

## Data Availability Statement

The data underlying the results presented in the study are available from the China Labor-force Dynamics Survey (CLDS). Anyone can access through application with the Center for Social Survey at Sun Yat-sen University at http://isg.sysu.edu.cn/node/425.

## Author Contributions

JinZ and JiaZ designed the model and the research frame work and wrote the manuscript. HZ contributed to the data preparation. JZha helped with the revision of literature review. All authors contributed to the article and approved the submitted version.

## Funding

This work was sponsored by the Social Sciences Foundation of Jiangsu Province under Grant Nos. 20SHC002 and 18GLD015, by the National Social Science Fund of China under Grant No. 20ARK003, and by the High-level Talent Program for Innovation and Entrepreneur of Jiangsu Province under Grant No. CZ0240619001.

## Conflict of Interest

The authors declare that the research was conducted in the absence of any commercial or financial relationships that could be construed as a potential conflict of interest.

## Publisher's Note

All claims expressed in this article are solely those of the authors and do not necessarily represent those of their affiliated organizations, or those of the publisher, the editors and the reviewers. Any product that may be evaluated in this article, or claim that may be made by its manufacturer, is not guaranteed or endorsed by the publisher.
